# Targeting CXCR4 with CTCE-9908 inhibits prostate tumor metastasis

**DOI:** 10.1186/1471-2490-14-12

**Published:** 2014-01-28

**Authors:** Donald Wong, Pridvi Kandagatla, Walter Korz, Sreenivasa R Chinni

**Affiliations:** 1 Department of Urology, Wayne State University School of Medicine, 9200 Scott Hall 540 E. Canfield Avenue, Detroit, MI 48201, USA; 2 Department of Pathology, Wayne State University School of Medicine, 9200 Scott Hall 540 E. Canfield Avenue, Detroit, MI 48201, USA; 3 The Barbara Ann Karmanos Cancer Institute, Detroit, MI 48201, USA; 4 British Canadian BioScience Corporation, Vancouver, Canada

**Keywords:** CTCE-9908, CXCR4, CXCL12, Chemoinvasion and prostate cancer progression

## Abstract

**Background:**

CXCL12/CXCR4 transactivation of epidermal growth factor family receptors in lipid raft membrane microdomains on cell surface is thought to mediate tumor growth and subsequent development of metastatic disease. CTCE-9908 is a known inhibitor of CXCR4. Herein, we tested the efficacy of CTCE-9908 in inhibiting prostate cancer cell growth, invasion, and metastasis.

**Methods:**

We used a panel of *in vitro* assays utilizing human prostate cancer cell lines and an *in vivo* orthotopic prostate cancer model to assess the anti-tumoral activity of CTCE-9908.

**Results:**

We demonstrated that (a) CTCE-9908 treatment resulted in no significant change in the growth of PC-3 and C4-2B cells; (b) 50 μg/ml of CTCE-9908 inhibited the invasive properties of PC-3 cells; (c) 25 mg/kg of CTCE-9908 did not alter primary tumor growth but it did significantly reduce total tumor burden in the animal including the growth of prostate and soft tissue metastases to lymph node and distant organ tissues. Histological analysis showed that CTCE-9908 treatment resulted in tumor necrosis in primary prostate tumors and no significant change in proliferation of tumor cells as measured by Ki-67 staining; (d) CTCE-9908 inhibited the tumor angiogenesis as measured by CD34 positive vessels in tumors.

**Conclusions:**

These data suggest that CXCR4 inhibition by CTCE-9908 decreases the invasion potential *in vitro*, which then translated to a reduction of tumor spread with associated reduction in angiogenesis. Hence, CTCE-9908 may prove to be an efficacious novel agent to prevent and treat the spread of metastatic prostate cancer.

## Background

CXCR4 activation contributes to site-specific metastasis in several types of tumors, where circulating epithelial tumor cells express CXCR4 and common metastasis sites express abundant ligand, and ligand/receptor interaction has been shown to promote metastasis (reviewed in [1]). In prostate cancer patients, CXCR4 expression is upregulated during cancer progression [2] and aggressive cancer development [3,4]. We and others have previously shown that the CXCL12/CXCR4 axis plays an important role in PC cell proliferation, migration, and invasion [2,5-13]. Furthermore, we demonstrated that CXCL12/CXCR4 signals through the PI3 kinase/Akt pathway to induce matrix metalloproteinase (MMP) expression and secretion, ultimately leading to migration and invasion of PC cells [5]. MMPs have been shown to be involved in the metastatic growth of prostate tumors in the bone and also appear to be activated at earlier time periods of tumor growth [14]; therefore, these data provide a mechanistic connection between “homing” of cancer cells to distant sites mediated by CXCL12/CXCR4 axis and followed by expression of MMPs which mediate invasion and proliferation. Similarly, our recent data demonstrate that CXCL12 binding to CXCR4 transactivates HER2 in PC cells to initiate chemo-invasive signaling and promotion of bone tumor growth, suggesting that this pathway is not only involved in initial seeding of bone metastases but also plays a key role in subsequent osseous expansion of metastases [10]. Furthermore, neutralization of CXCR4 in prostate cancer cells with anti-CXCR4 antibodies significantly reduced metastatic burden of experimental bone metastasis [13].

Targeting CXCR4 can have dual effects on inhibiting primary tumor growth and metastasis or mono effect on inhibiting either tumor growth or metastasis. Among CXCR4 inhibitors, AMD3100 is in clinical use for leukemia [15,16], and CTCE-9908 was granted approval by the FDA for osteosarcoma [17] based on its potent inhibitory activity in preclinical models of osteosarcoma [18]. AMD3100 is a bicyclam CXCR4 inhibitor that has been shown to be effective in reducing tumor growth in glioblastoma [19] and peritoneal metastasis in ovarian carcinoma [20]. CTCE-9908 is a peptide antagonist for CXCR4 and has shown to inhibit both primary tumor growth and metastases in osteosarcoma [18] and breast cancer models [21]. In a prostate cancer model, CTCE-9908 caused a reduction in tumor growth in a subcutaneous xenograft model via inhibiting angiogenesis by reducing the recruitment of pro-angiogenic myeloid precursor cells [22]. The current study assessed the efficacy of CTCE-9908 in an orthotopic prostate cancer model of primary tumor growth and metastases. The results show that CTCE-9908 is effective in reducing total tumor burden without significantly affecting the primary tumor growth.

## Methods

### Cell culture

PC-3 cells were obtained from American Type Culture Collection (Manassas, VA) and cultured in RPMI 1640 (Invitrogen Life Technologies, Carlsbad, CA) supplemented with 10% FBS and 1% Penicillin and Streptomycin. C4-2B cells were obtained from Dr. Leeland Chung and cultured in T media (Invitrogen Life Technologies, Carlsbad, CA) supplemented with 10% FBS and 1% Penicillin and Streptomycin.

### Cell proliferation assay

1×10^4^ PC-3 and C4-2B cells were seeded in a 96 well plate; the following day, cells were treated with varying concentrations (0–100 μg/ml) of CTCE-9908 dissolved in sterile dH_2_O. After 24, 48, and 72 hours, cells were washed with PBS and exposed to 1X dye binding solution from CyQUANT® NF cell proliferation assay kit (Molecular Probes, Eugene, OR) for 60 min. Dye DNA-bound complexes were measured at 485 nm excitation and 530 nm emission.

### Chemoinvasion assay

PC-3 cells were serum-starved for 4 hours. A total of 1.5-2.0 × 10^5^ cells were seeded onto inserts in the upper chamber of trans-well culture plates (Becton Dickenson, San Diego, CA). Prior to seeding, the inserts were pre-coated with Matrigel. Untreated control and CTCE-9908 (1 and 50 μg/ml) pretreated PC-3 cells were seeded in Matrigel coated inserts. CXCL12 was placed in the bottom chamber to induce CXCR4-mediated chemoinvasion. After 24 hours, the upper chambers were cleaned with cotton swabs to remove non-migrated/invaded cells, and the inserts were stained with Diff-Quik stain set (Dade Behring Inc., Newark, DE). The total number of migrated cells in a high power field was counted under a microscope, and the data presented is based on three independent experiments.

### Orthotopic tumor growth and CTCE-9908 treatment

PC-3-GFP cells were grown subcutaneously as a tumor stock. The animal experiments were performed at Anti Cancer Inc., (San Diego, CA) in accordance with the principles and procedures outlined in the NIH Guide for the Care and Use of Laboratory Animals under assurance number A3873-1. Subcutaneous PC3-GFP tumor was excised, the necrotic areas removed, and 1 mm^3^ of tumor piece was implanted in the mouse ventral lobe of the prostate [23]. Animals were treated with 25 mg/kg/day CTCE-9908 daily, through subcutaneous injection at 3 days post-orthotopic tumor implantation for four weeks. Control animals were treated with water. The whole experiment was performed in three batches with six, four and ten animals in control group to a total of 20 mice and seven, four and ten animals in CTCE-9908 group to a total of 21 mice. Four weeks post tumor implantation, caliper measurements were made to determine the orthotpic prostate tumor volume by measuring perpendicular minor dimension (W) and major dimension (L) and volume was calculated by the W^2^ × L × 1/2 formula. Whole body fluorescence measurements were made using Leica stereo fluorescence microscope (model LZ12) equipped with ST-133 Micromax high speed CCD camera (Princeton Instruments, Trenton, NJ) with anesthetized intact animals to determine total tumor burden including metastases. Starting two weeks after implantation whole body imaging was performed once a week. At the end of the study (four weeks) animals were euthanized and an open imaging was conducted to accurately document and quantitate tumor burden (primary tumors and metastatic tumor). Signals from individual organ metastasis were quantitated from control animals and CTCE-9908 treated animals.

### Immunohistochemistry

Prostate tumors and lymph node metastases were paraffin embedded, and 4 μm thick sections were cut using microtome. Tissue sections were immunostained with anti-cytokeratin antibody, anti-Ki-67 antibody and anti-CD34 antibody. Vectastain Elite ABC kit was used to stain secondary antibody, and DAB chromagen was used to develop color. Images were captured with Axiovision software. For determining angiogenesis clusters of CD34 positive vessels (hot spots) were counted in 400× field in tumors and lymph node metastasis.

### Statistical analysis

*In vitro* studies, statistical significance was determined by Student t-test and non-parametric ANOVA test, while in vivo assays were analyzed by Student t-test using GraphPad Prism software version 3.0 (GraphPad, San Diego, CA). *p* ≤0.05 was considered to be statistically significant.

## Results

### CXCR4 inhibition by CTCE-9908 does not inhibit cell proliferation

**Figure 1:**
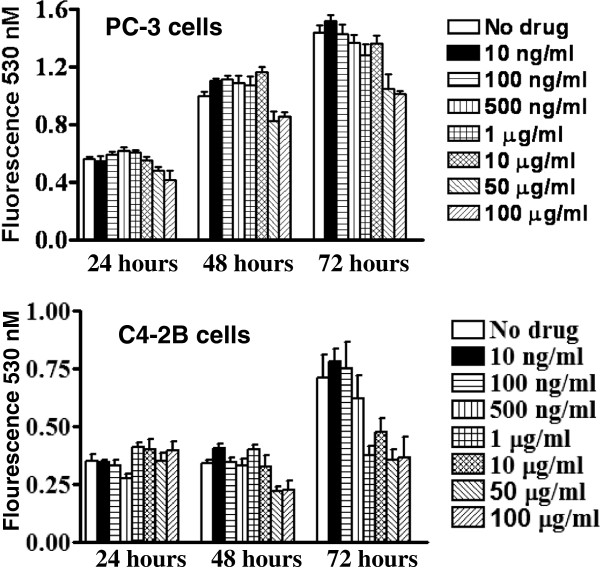
**PC-3 cell proliferation in the presence of CTCE-9908.** PC-3 and C4-2B cells were treated with 0, 10, 100, 500 ng/ml, 1, 10, 50 and 100 μg/ml concentrations of CTCE-9908 for 24 to 72 hours, and viable cells were determined with CyQUANT® NF cell proliferation assay.

In an effort to determine the effect of CTCE-9908 on proliferation, PC-3 and C4-2B cells were treated with increasing concentrations of CTCE-9908 ranging from 10 ng/ml to 100 μg/ml for 24 to 72 hours. CTCE-9908 treatment did not significantly affect the PC-3 cell proliferation (Figure 1). Similar trend was observed with C4-2B cells up to 48 treatment of CTCE-9908, but at 72 hours a modest inhibition of growth observed at higher concentrations of CTCE-9908.

### CTCE-9908 inhibits CXCL12-induced cell invasion

**Figure 2:**
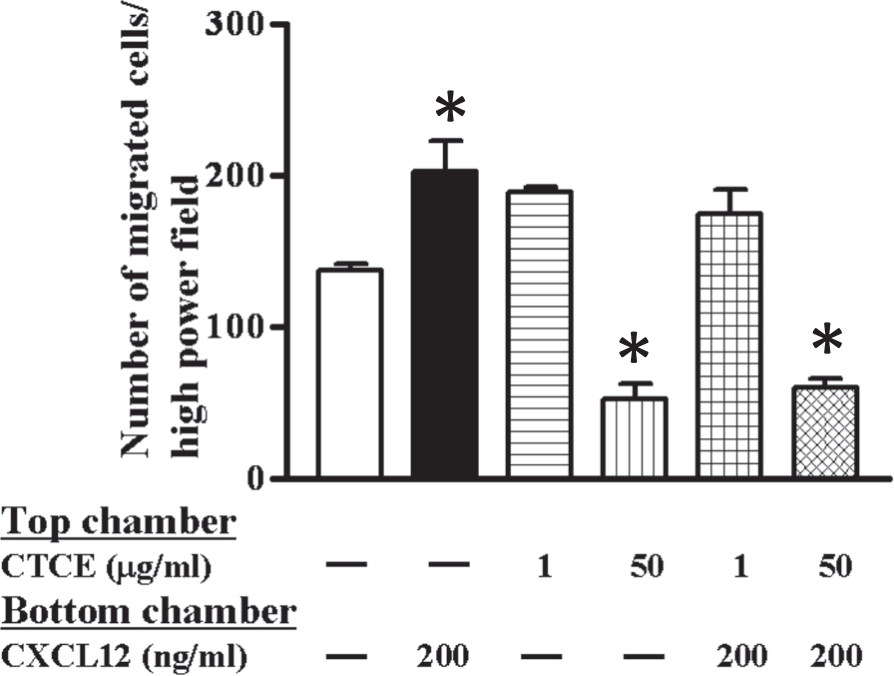
**PC-3 cell chemoinvasion in the presence of CTCE-9908.** Chemonivasion of PC-3 cells were performed with Matrigel-coated inserts. PC-3 cells were pretreated with 1 and 50 μg/ml of CTCE-9908 for 48 hours then seeded in chemoinvasion inserts and exposed to CTCE-9908. 200 ng/ml of CXCL12 was added to the lower chamber as a chemoattractant. Invaded cells at the bottom of the filter were quantitated and shown in figure.

Our previous reports demonstrate that CXCL12/CXCR4 activation induces protease expression and chemoinvasion of PC-3 cells. Herein, the effect of CTCE-9908 on CXCL12-induced chemoinvasion was tested in PC-3 cells as the drug have no growth inhibitory effect. As expected, CXCL12 induced chemoinvasion of PC-3 cells; treatment with 50 μg/ml CTCE-9908 significantly reduced CXCL12-induced chemoinvasion of PC-3 cells (Figure 2). These results suggest that CTCE-9908 compound inhibits the CXCL12/CXCR4 axis and subsequent chemoinvasion of PC-3 cells.

### CXCL12/CXCR4 inhibition by CTCE-9908 leads to inhibition of total tumor burden

**Figure 3:**
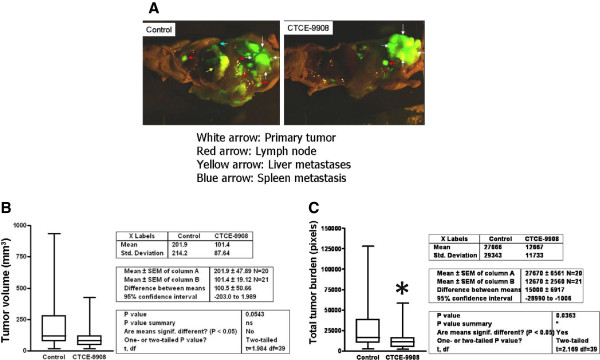
**CTCE-9908 intervention studies with PC-3 orthotropic prostate tumors.** PC-3 tumors were grown in mouse prostate through orthotropic implantation. 25 mg/kg of CTCE-9908 was administered to the mice. **A)** Whole body GFP fluorescence image of mice. **B)** Prostate tumor volume was measured by calipers. **C)** Total GFP fluorescence of mice.

To determine the efficacy of CTCE-9908 in inhibiting CXCL12/CXCR4-mediated tumor cell growth and dissemination, an orthotopic model of prostate cancer metastasis was utilized. GFP-transfected PC-3 tumor cells were implanted into the ventral lobes of murine prostate. GFP stable transfection did not affect CXCR4 expression (data not shown). Mice were treated with a daily dose of 25 mg CTCE-9908/kg mouse body weight for four weeks. Caliper measurements of tumor volume at the end of four weeks show a decrease in mean tumor volume in CTCE-9908 treated animals (Figure 3B), although this decrease was not statistically significant. Proliferation index was determined in control and CTCE-9908 treated prostate tumors by immunostaining tumor sections for Ki-67 expression. There is no significant difference present between Ki-67 positive tumor cells between these groups suggesting that proliferation rate of tumor cells was not affected by the CTCE-9908 (Additional file 1: Figure S1). The reduction in tumor growth may be due to increased necrosis of tumor cells as evidenced by low cytokeratin staining in CTCE-9908 treated mice (Additional file 2: Figure S2) which resulted in shrinkage of tumor.

CTCE-9908 treatment significantly reduced lymph node metastasis and distant metastases in orthotopic mouse model (Figure 3A), however significant reduction in primary tumor burden was not evident (Figure 3B). Total body fluorescence measurements show that CTCE-9908 treatment significantly inhibited total metastatic burden in mice (Figure 3C). Quantitation of site-specific metastases show that lymph node metastases were reduced by 40%, spleen metastasis by 75%, liver metastasis by 93%, and 95% reduction in distant metastases in CTCE-9908 treated mice (data not shown). Taken together, these data demonstrate that CTCE-9908 administration significantly inhibited dissemination of cancer cells to various sites in the mouse.

### CTCE-9908 inhibits angiogenesis in prostate tumor tissues

**Figure 4:**
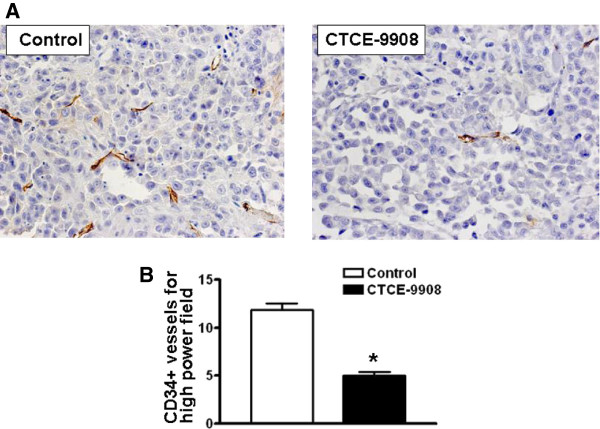
**Microvessel density in CTCE-9908-treated PC-3 tumors. A)** Prostate tumor tissues were stained with anti-CD 34 antibody. **B)** Quantitation of CD34+ vessels in control and CTCE-9908 treated prostate tumor tissue. *Represents statistically significant, where p = 0.0021.

Primary tumor tissue from control and CTCE-9908-treated mice were stained with anti-CD34 antibody to determine the effect of CTCE-9908 on tumor angiogenesis. As shown by immunohistochemistry, CTCE-9908 treated tumors have fewer vessels, and these vessels are also smaller in size (Figure 4A). Quantitation of microvessel density in the hot spots of angiogenesis show a reduced CD34-positive vessel density in CTCE-9908 treated tumors (Figure 4B). Moreover, quantitation of CD34 positive vessel density in lymph node metastatic tissue shows a decrease in number in CTCE-9908 treated tumors (Additional file 3: Figure S3). These data suggest that CTCE-9908 treatment inhibited the angiogenesis of primary and lymph node metastatic tumors. The CTCE-9908-mediated inhibition of primary tumor angiogenesis lead to inhibition of metastasis.

## Discussion

Previous studies demonstrate that tumor cells are capable of usurping immune cell chemoinvasive pathways for metastasis to secondary sites. Chemokines and chemokine receptors mediate physiological movement of immune cells in the body. Among the family of chemokine and chemokine receptors mediating tumor cell invasion and metastasis, CXCL12/CXCR4 has gained a central role in different types of tumors in mediating tumor growth, angiogenesis and metastasis. In prostate cancer cells, CXCL12 and CXCR4 play a key role in invasion and metastasis, leading to development and expansion of osseous metastasis. In this study we assessed the effect of inhibition of the CXCL12/CXCR4 pathway by a novel CXCR4 antagonist, CTCE-9908 on *in vitro* cell proliferation and invasion, and *in vivo* orthotopic tumor growth, metastasis, and angiogenesis of PC cells.

Previous studies report that CTCE-9908 compound inhibited cell proliferation in PC-3 cells at higher concentrations with no effect at lower concentrations [22]; our data is in line with these studies, as CTCE-9908 compound did not show significant inhibition in cell proliferation at 100 μM (which corresponds to 44 nM) concentration. This lack of inhibitory effect on PC-3 cells can be attributed to the fact that cultured PC-3 cells express low or no CXCL12 [5], and therefore CXCR4 activation could be low in these cells. Previous report by Provasnik *et al.* support this observation that CTCE-9908 administration do not inhibit the subcutaneous tumor growth [22]. As opposed to cultured cancer cells, *in vivo* bone tumors express CXCL12 in prostate cancer cells in addition to osteoblasts and endothelial cells. Primary tumors also express CXCL12 in epithelial cells. The CXCL12/CXCR4 axis has been shown to promote cell survival by inhibiting apoptosis in cancer cells; thus, CTCE-9908-mediated inhibition of the CXCL12/CXCR4 pathway leads to loss of protection from apoptosis and increased cell death. Our data support this notion, as CTCE-9908-treated tumors showed enhanced necrotic areas, suggesting that loss of the CXCL12/CXCR4 axis mediated cell survival leading to enhanced necrosis in tumor cells. But, we cannot rule out the role of growth inhibition of CTCE-9908 in our model as mean tumor growth is inhibited in CTCE-9908 treated group, though the data did not reach statistical significance.

We have previously shown that the CXCL12/CXCR4 axis in PC-3 cells induce MMP-9 expression via activation of PI3K and MAPK pathways, and this activation mediates *in vitro* cell invasion of PC-3 cells [24]. Bone colonizing PC-3 cells induce the expression of active MMP-9 at earlier time periods suggesting that CXCL12/CXCR4-mediated homing of PC cells to bone would functionally link with the expression of MMP-9 in local bone tumor microenvironment and induce invasive bone tumor growth [5]. To determine whether CTCE-9908 compound could inhibit invasion of PC-3 cells, we used the lower concentration of 50 μg/ml in cell invasion studies. Although this concentration of CTCE-9908 did not inhibit cell proliferation, our data suggest that 50 μg/ml CTCE-9908 potently inhibited the CXCL12-induced PC-3 cell invasion. To determine whether inhibition of invasion could translate into inhibition of metastasis formation, we treated mice implanted with orthotopic tumors with CTCE-9908. The whole body quantitation of fluorescence measurements shows that CTCE-9908 treatment significantly reduced total tumor burden as a measure of total body fluorescence. To our knowledge, this is the first report to document that targeting the CXCL12/CXCR4 axis through CTCE-9908 inhibited the metastatic burden in an orthotopic prostate cancer model system. Both lymph node and distant metastases were significantly inhibited in CTCE-9908 treated tumors, but distant metastases were strongly inhibited compared to lymph node metastases. Similar observations were found with CTCE-9908 in a breast cancer model where total metastatic burden was significantly inhibited upon CTCE-9908 administration [25]. CXCL12/CXCR4 mediated invasive function has implications in clinical management of patients as chemotherapy resistant tumors cells often express high levels of CXCR4 [26] and this may lead to the development of metastases in these patients via CXCL12/CXCR4 activation. In addtion, prostate cancer progenitor cells express CXCR4 [27] and often these cells are resistant to current chemo and radiation therapy practices, thus, combination therapy with anti-CXCR4 strategies consisting of CTCE-9908 may prevent the further spread of tumor in patients.

Tumor angiogenesis plays a key role in tumor growth and development of metastases. CXCL12/CXCR4 signaling has been shown to modulate the expression of angiogenic cytokines/chemokines in prostate cancer cells [28]. Expression of these proangiogenic factors can recruit endothelial precursor cells to the tumor sites to facilitate angiogenesis. To determine the effect of CXCR4 inhibition on tumor angiogenesis we measured hotspots of angiogenesis in primary and lymph node metastatic tumor tissues for CD34 positive blood vessels. CTCE-9908 treatment significantly inhibited angiogenesis in both primary and lymph node metastases. Porvasnik et al. reported that CTCE-9908 treatment reduced tumor angiogenesis by down regulating VEGF production and myeloid derived suppressor cell (CD11b positive) recruitment into tumor tissues [22]. CD11b cells have been recently shown to express CXCR4 and migrate towards the CXCL12 expressing cells.

Our studies show that CTCE-9908 is efficacious in inhibiting total tumor burden without significantly reducing primary tumor burden suggesting that targeting CXCL12/CXCR4 axis may be therapeutically beneficial for the management of prostate cancer patients undergoing chemo or radiation therapy.

## Conclusions

The data presented in the study demonstrate that CTCE-9908 is efficacious in preventing spread of tumor cells from primary site by inhibiting invasive and angiogenic functions of CXCL12/CXCR4 axis in primary tumor environment.

## Competing interests

Dr. Donald Wong and Mr. Walter Korz are the employees of British Canadian BioScience Corporation.

## Authors’ contributions

DW and WK participated in study design and acquisition of animal experimental data. PK carried out immunohistochemical experiments and involved in preparation of figures. SRC is actively involved in all aspects of study and responsible for drafting manuscript. All authors read and approved the final manuscript.

## Pre-publication history

The pre-publication history for this paper can be accessed here:

http://www.biomedcentral.com/1471-2490/14/12/prepub

## Supplementary Material

Additional file 1: Figure S1 **A)** Immunohistochemical analysis of Ki-67 in PC-3 tumors. Control and CTCE-9908 treated prostate tumor tissues were stained for Ki-67 antigen. **B)** Quantitation of total Ki-67 positive cells in control and treated group. Statistical difference between groups is not significant, where p = 0.0897. Click here for file

Additional file 2: Figure S2 Immunohistochemical analysis of cytokeratin in PC3 tumors. Control and CTCE-9908 treated prostate tumor tissues were stained for cytokeratin. Arrow represents low staining necrotic area. Click here for file

Additional file 3: Figure S3 CD34 staining of lymph node metastasis in control (left) and CTCE-9908 treated mice (right). A representative hot spot of CD34+ vessels is shown. Graphical representation of microvessel densities between control and treated groups of metastatic tumor sections. *represents statistically significant, where p = 0.0296. Click here for file
