# Supplementary figures and images for: Targeting CXCR4 with CTCE-9908 inhibits prostate tumor metastasis
Source: BMC Urol. 2014 Jan 28;14:12. doi: 10.1186/1471-2490-14-12 (PMC3912255; doi:10.1186/1471-2490-14-12)

## Slide 1
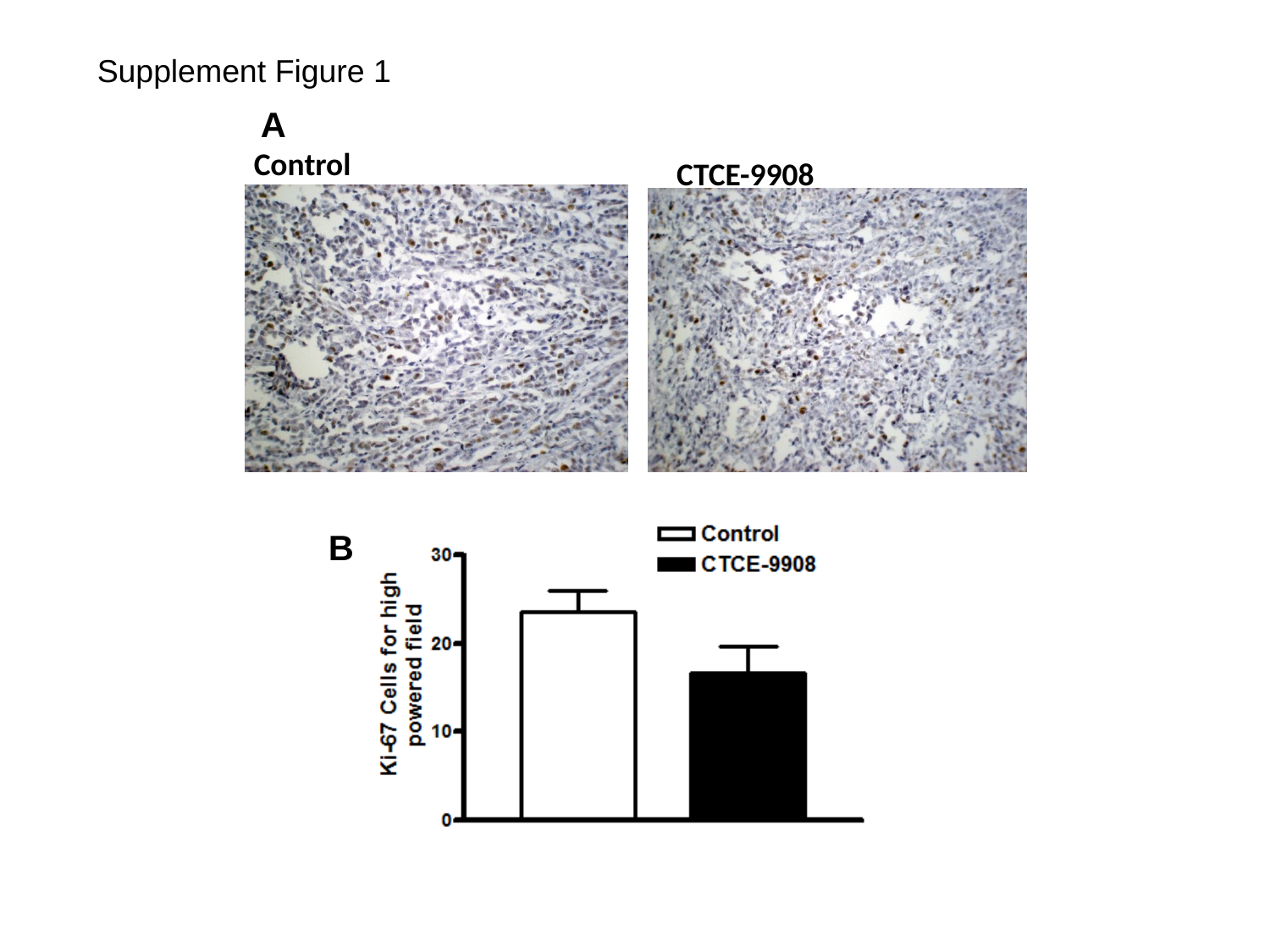

Supplement Figure 1
A
Control
CTCE-9908
B

Supplement: Additional file 1: Figure S1 — A) Immunohistochemical analysis of Ki-67 in PC-3 tumors. Control and CTCE-9908 treated prostate tumor tissues were stained for Ki-67 antigen. B) Quantitation of total Ki-67 positive cells in control and treated group. Statistical difference between groups is not significant, where p = 0.0897. [file 1471-2490-14-12-S1.pptx]

## Slide 1
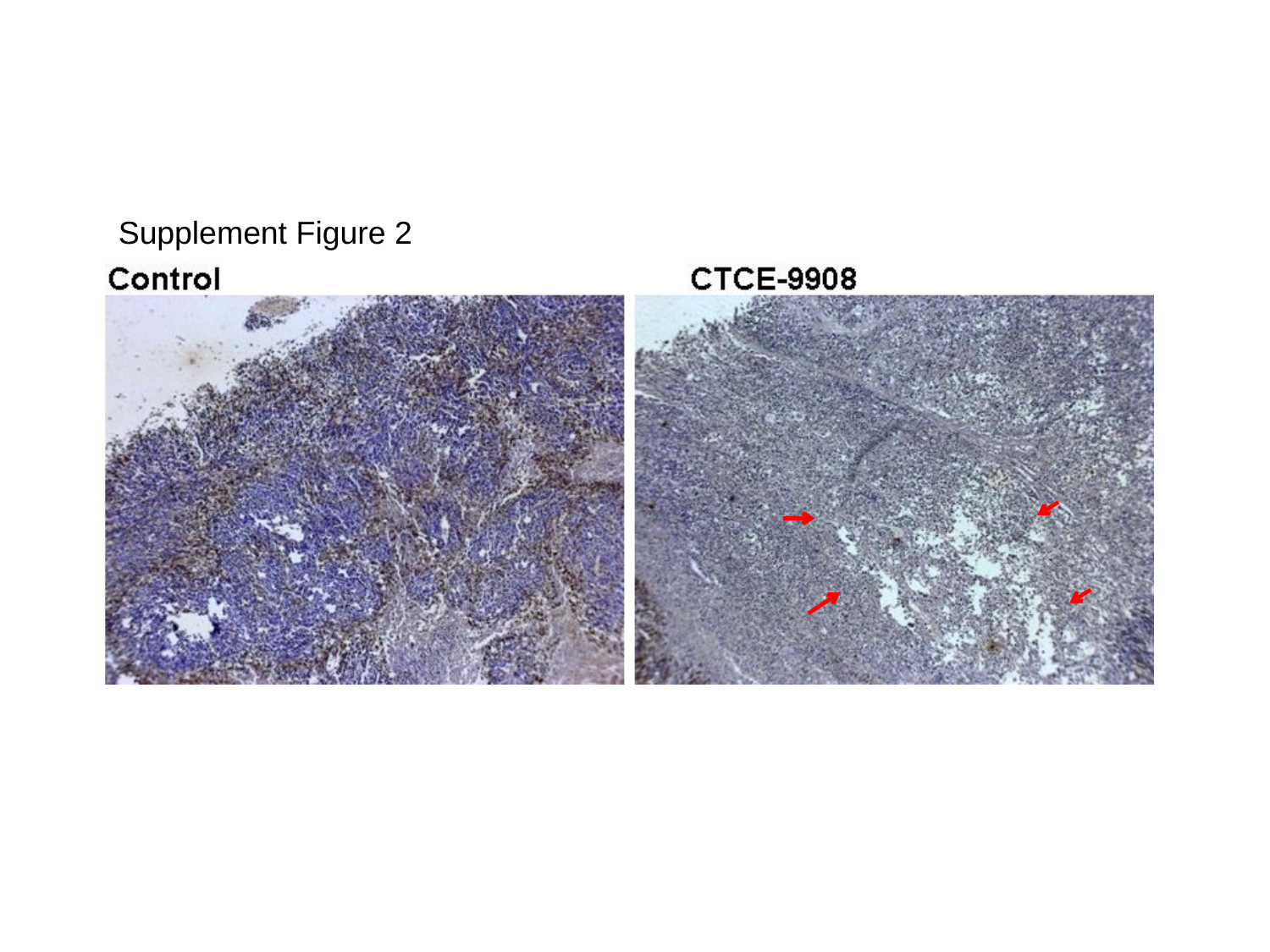

Supplement Figure 2

Supplement: Additional file 2: Figure S2 — Immunohistochemical analysis of cytokeratin in PC3 tumors. Control and CTCE-9908 treated prostate tumor tissues were stained for cytokeratin. Arrow represents low staining necrotic area. [file 1471-2490-14-12-S2.pptx]

## Slide 1
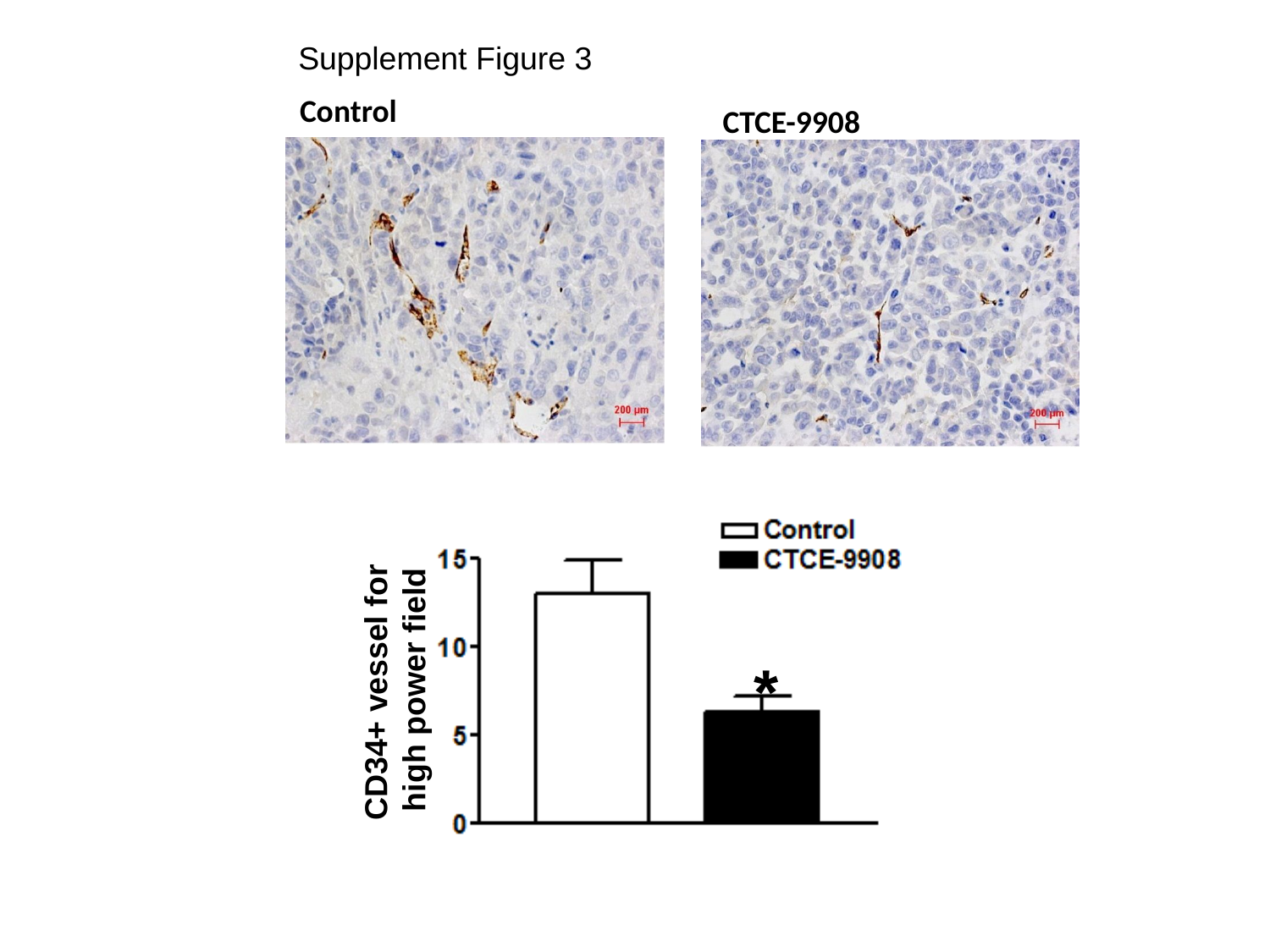

Supplement Figure 3
Control
CTCE-9908
*
CD34+ vessel for
 high power field

Supplement: Additional file 3: Figure S3 — CD34 staining of lymph node metastasis in control (left) and CTCE-9908 treated mice (right). A representative hot spot of CD34+ vessels is shown. Graphical representation of microvessel densities between control and treated groups of metastatic tumor sections. *represents statistically significant, where p = 0.0296. [file 1471-2490-14-12-S3.pptx]
